# Associations between Infant Feeding Practice Prior to Six Months and Body Mass Index at Six Years of Age

**DOI:** 10.3390/nu6041608

**Published:** 2014-04-17

**Authors:** Cindy Mari Imai, Ingibjorg Gunnarsdottir, Birna Thorisdottir, Thorhallur Ingi Halldorsson, Inga Thorsdottir

**Affiliations:** 1 Unit for Nutrition Research, Landspitali—National University Hospital of Iceland, Eiriksgata 29, Reykjavik 101, Iceland; E-Mails: ingigun@landspitali.is (I.G.); bth50@hi.is (B.T.); tih@hi.is (T.I.H.); ingathor@hi.is (I.T.); 2 Faculty of Food Science and Nutrition, School of Health Sciences, University of Iceland, Eiriksgata 29, Reykjavik 101, Iceland

**Keywords:** MeSH terms, growth, infant, breastfeeding, weaning, overweight, child

## Abstract

Rapid growth during infancy is associated with increased risk of overweight and obesity and differences in weight gain are at least partly explained by means of infant feeding. The aim was to assess the associations between infant feeding practice in early infancy and body mass index (BMI) at 6 years of age. Icelandic infants (*n* = 154) were prospectively followed from birth to 12 months and again at age 6 years. Birth weight and length were gathered from maternity wards, and healthcare centers provided the measurements made during infancy up to 18 months of age. Information on breastfeeding practices was documented 0–12 months and a 24-h dietary record was collected at 5 months. Changes in infant weight gain were calculated from birth to 18 months. Linear regression analyses were performed to examine associations between infant feeding practice at 5 months and body mass index (BMI) at 6 years. Infants who were formula-fed at 5 months of age grew faster, particularly between 2 and 6 months, compared to exclusively breastfed infants. At age 6 years, BMI was on average 1.1 kg/m^2^ (95% CI 0.2, 2.0) higher among infants who were formula fed and also receiving solid foods at 5 months of age compared to those exclusively breastfed. In a high-income country such as Iceland, early introduction of solid foods seems to further increase the risk of high childhood BMI among formula fed infants compared with exclusively breastfed infants, although further studies with greater power are needed.

## 1. Introduction

Childhood obesity is an ongoing public health concern [1]. Infant growth patterns are becoming better understood and the timing and tempo of growth rate appears to be important variables in predicting childhood obesity [2,3]. Rapid growth during the first year of life has been associated with increased risk of overweight and obesity in children [4,5] and differences in weight gain during early infancy are at least partly explained by means of infant feeding [6,7,8].

The World Health Organization (WHO) recommends exclusive breastfeeding for the first 6 months of life and its benefits are well accepted worldwide [6,9]. However, there is evidence that most mothers in European countries begin to introduce complementary foods before 6 months of age [10]. Studies have shown that formula fed children grow more rapidly than children who are exclusively breastfed [11,12] and are at greater risk of childhood overweight or obesity [6] but less is known about the long-term effects of early solid food introduction in early infancy. In a recent randomized controlled trial, introduction of complementary feeding at 4 months of age did not increase weight gain in infancy and did not appear to affect the risk of overweight or obesity at 18 months or 29–38 months of age compared to infants exclusively breastfed for 6 months [13,14,15]. The aim in this current analysis was to assess the associations between infant feeding practice in early infancy and body mass index (BMI) at 6 years of age. Furthermore, we aimed to assess whether the introduction of solid foods among partially breastfed or formula fed infants prior to 6 months of age was associated with childhood BMI.

## 2. Experimental Section

### 2.1. Study Design

The study population, recruitment and data collection have previously been described in detail [16]. In brief, a random sample of 250 Icelandic infants born in 2005 was collected by Statistics Iceland. The inclusion criteria were Icelandic parents, singleton birth, gestational length of 37–41 weeks, birth weight within the 10th and 90th percentiles, no birth defects or congenital long-term diseases, and the mother had early and regular antenatal care. In this current analysis, eligible subjects were those with complete data at birth and with a complete dietary record at 5 months, and weight and height measurements at 6 years of age (*n* = 154). Our analysis are mainly based on the feeding practice at the age of 5 months where we compare the growth in infancy between those children who were exclusively breastfed at this time point to those who were either exclusively formula fed or those who had started complementary feeding (defined here as introduction of solid foods in addition to partial breastfeeding or formula feeding) at the age of 5 months. The reason why we chose to use the 5 month registration for our primary analysis is that this was the earliest detailed food registration available in the present study and it gives information on variations in duration of exclusive breastfeeding. Informed written consent was obtained from all parents. The study was approved by the Icelandic Data Protection Authority (S5099/2011), Local Ethical Committee at Landspitali-University Hospital (1104Ref.16 2011) and the Bioethics committee (VSNb2011010008/037).

### 2.2. Infant and Childhood Growth Data Collection

Birth information on weight and length was gathered from the maternity wards. Healthcare centers provided the measurements made during infancy at 1, 2, 3, 5, 6, 8, 10, 12 and 18 months of age. As close to the child’s sixth birthday as possible (mean 73.4 ± 3.2 months) weight (Marel M series 1100, Iceland; ±0.1 kg) and height (Ulmer stadiometer, Professor Heinze, Busse design, Ulm, Germany; ±0.5 cm) were measured in a clinical examination at the Landspitali-University Hospital. BMI was calculated as weight (kg)/height (m^2^). When the infant was 12 months of age, the parent or caregiver was asked to complete a questionnaire regarding information on age, education, and physical characteristics including self-reported height and weight of both parents.

### 2.3. Dietary Assessment

Information on breastfeeding was gathered monthly during the first 12 months. The parents or caregivers completed a 24-h food record monthly from 5 to 8 months and 10 to 11 months using common household measures such as cups and spoons. At 9 and 12 months of age, weighed food records were kept for three consecutive days on accurate scales (PHILIPS HR 2385, Austria; PHILIPS HR 2385, Hungary; ±1 g accuracy).

Average daily consumption of energy and the contribution of energy providing nutrients at the age of 9 and 12 months were estimated using ICEFOOD, a software program used by the Icelandic Nutrition Council. Special infant products, such as cereals and purées were added to the database and nutrient losses due to food preparation were taken into account in the calculations.

### 2.4. Statistical Analysis

Mean and standard deviation (SD) or proportions (%) were used to describe infant and maternal characteristics. Differences in infant and maternal characteristics were calculated using *t*-tests for continuous variables and chi-squared tests for categorical variables. Linear regression analyses were used to determine associations between infant feeding practice at 5 months of age and BMI at 6 years. All regression analyses were adjusted for sex, birth weight, and maternal education level categorized as completion of elementary school, high school or vocational school, or university. Information on mother’s education was missing for *n* = 12, thus a total of 142 subjects were analyzed for the linear regression. Changes in growth were calculated from crude differences in measurements at the two different time points. All statistical analyses were carried out using SPSS version 20.0 (IBM Corp., Armonk, NY, USA). The level of significance was set at *p* < 0.05.

## 3. Results

Table 1 shows the participants’ characteristics among infants at 5 months of age who were exclusively breastfed (*n* = 62), exclusively formula fed (*n* = 12), started on solid foods with partial breastfeeding (*n* = 57) or started on solid foods with formula feeding (*n* = 23).

**Table 1:** Participant characteristics and dietary variables by infant feeding practice at 5 months of age.

Infant Feeding Practice at 5 Months				
Variable	Exclusively Breastfed (*n* = 62)	Excusively Formula Fed (*n* = 12)	Solid Foods with Partial Breastfeeding (*n* = 57)	Solid Foods with Formula Feeding (*n* = 23)
	Mean ± SD	Mean ± SD	Mean ± SD	Mean ± SD
*Maternal characteristics*				
Age (years)	31.5 ± 4.5	30.4 ± 4.0	31.0 ± 4.8	30.1 ± 4.8
BMI (kg/m^2^)	23.7 ± 3.2	29.2 ± 9.1	24.5 ± 3.8 *	27.0 ± 6.5
University education [*n* (%)]	31 (50)	8 (67)	28 (49)	6 (26)
*Infant characteristics*				
Girls [*n* (%)]	34 (55)	24 (55)	24 (42)	14 (61)
Exclusive breastfeeding (month)	5.0 ± 0.9	1.7 ± 1.7 *	3.1 ± 1.5 *	1.4 ± 1.5 *
Breastfeeding duration (month)	9.5 ± 1.9	3.9 ± 2.7 *	8.3 ± 2.4 *	2.8 ± 1.1 *
*Food groups introduced at 5 months*				
Porridge [*n* (%)]	-	-	53 (93)	18 (78)
Fruits and vegetables [*n* (%)]	-	-	20 (35)	15 (65)
Dairy products [*n* (%)]	-	-	20 (35)	17 (74)
Legumes [*n* (%)]	-	-	1 (2)	3 (13)
Meat, organs [*n* (%)]	-	-	0	0
Eggs [*n* (%)]	-	-	0	0
*Dietary composition at 9 months*				
Energy (kcal/kg)	101 ± 36	109 ± 27	101 ± 31	101 ± 48
Protein (g/kg)	3.0 ± 1.4	3.7 ± 1.4	3.2 ± 1.4	3.6 ± 1.7
*Dietary composition at 12 months*				
Energy (kcal/kg)	89 ± 22	80 ± 16	86 ± 18	79 ± 16
Protein (g/kg)	3.1 ± 0.9	3.0 ± 0.8	3.6 ± 1.7	3.2 ± 0.9

* Significantly different from exclusively breastfed infants determined by *t*-tests for continuous variables and chi-square tests for categorical variables.

Maternal characteristics were not markedly different although mothers who exclusively breastfed their child at 5 months had lower BMI. The total duration of breastfeeding was shortest among infants who were formula fed (both with and without solid food introduction) at the age of 5 months. Among infants who were provided solid foods at 5 months, the food items with the highest frequency of consumption were porridge, dairy products, and fruits and vegetables. Based on the 24-h dietary registration at 5 months, there were no children eating meat, fish, poultry, liver, or eggs although it is possible they were exposed to these foods. Although no difference was observed in energy intake at 9 or 12 months based on infant feeding practice at the age of 5 months, infants who were receiving formula feeds had higher protein intake per kilogram at 9 months.

There were no differences in birth weight between the different infant feeding groups (Table 2). However, infants who were formula fed at 5 months grew faster after birth, particularly between 2 and 6 months of age compared to exclusively breastfed infants (Table 3).

**Table 2:** Anthropometrics from birth by infant feeding practice at 5 months of age.

Growth Variable	Exclusively Breastfed (*n* = 62)	Exclusively Formula Fed (*n* = 12)	Solid Foods with Partial Breastfeeding (*n* = 57)	Solid Foods with Formula Feeding (*n* = 23)
	Mean ± SD	Mean ± SD	Mean ± SD	Mean ± SD
Weight (kg)				
At birth	3.8 ± 0.4	3.8 ± 0.4	3.7 ± 0.4	3.7 ± 0.3
2 months	5.6 ± 0.6	5.5 ± 0.7	5.8 ± 0.5	5.4 ± 0.4
6 months	7.8 ± 0.8	8.2 ± 1.0	8.2 ± 1.0	8.5 ± 0.8 *
2 months	9.6 ± 0.9	10.3 ± 1.4	10.1 ± 1.2 *	10.6 ± 1.0 *
18 months	11.3 ± 1.0	11.5 ± 1.2	11.6 ± 1.3	11.9 ± 1.2 *
Length (cm)				
At birth	51.9 ± 1.5	52.0 ± 1.7	51.8 ± 1.6	51.7 ± 1.9
2 months	59.7 ± 1.5	59.4 ± 1.8	59.8 ± 2.0	58.9 ± 1.5
6 months	68.3 ± 2.0	69.4 ± 2.7	69.1 ± 2.3 *	68.4 ± 1.7
12 months	76.2 ± 2.5	77.4 ± 2.9	77.1 ± 2.7	77.6 ± 2.5 *
18 months	83.0 ± 2.5	83.8 ± 3.2	83.9 ± 2.7	83.1 ± 2.5

* Significantly different from exclusively breastfed infants determined by *t*-tests.

**Table 3:** Changes in weight from birth to 18 months by infant feeding practice at 5 months comparing infants on formula or solid foods, Δ (95% CI), to exclusively breastfed infants.

Weight Variable	Exclusively Breastfed (referent) (*n* = 62)	Exclusively Formula Fed (*n* = 12)	Solid Foods with Partial Breastfeeding (*n* = 57)	Solid Foods with Formula Feeding (*n* = 23)
Changes in weight (g)	Mean ± SD	∆ (95% CI) ^a^	∆ (95% CI) ^a^	∆ (95% CI) ^a^
Birth to 2 months	1859 ± 500	−188 (−531, 155)	156 (−31, 343)	−119 (−369, 132)
2 to 6 months	2208 ± 480	512 (147, 877) *	161 (−63, 385)	787 (472, 1102) *
6 to 12 months	1775 ± 550	172 (−193, 538)	122 (−85, 328)	283 (−8, 574)
12 to 18 months	1649 ± 563	−56 (−450, 338)	−90 (−297, 116)	−314 (−634, 6)
Birth to 6 months	4081 ± 745	308 (−168, 785)	360 (58, 661) *	755 (367, 1143) *
Birth to 12 months	5876 ± 884	510 (−108, 1129)	462 (92, 831) *	1019 (566, 1471) *

^a^ Mean difference in weight compared to exclusively breastfed infants (referent); * Significantly different from exclusively breastfed infants determined by *t*-tests.

Furthermore, the group that had been introduced to solid foods along with formula feeding at the age of 5 months grew significantly faster compared to exclusively breastfed infants, while the difference was smaller and non-significant for the group who had been provided solid foods and were partially breastfed (Table 3). The addition of solid foods along with formula feeding at 5 months predicted greater BMI at 6 years, with BMI being on average 1.1 kg/m^2^ (95% CI 0.2, 2.0) higher compared to exclusively breastfed infants at 5 months of age (Table 4).

**Table 4:** Associations between infant feeding practice at 5 months and BMI at 6 years of age (*n* = 142).

Infant Feeding Practice at 5 Months	∆ (95% CI) ^a^	*p* *
Exclusively breastfed	Referent	*-*
Formula fed	0.3 (−0.8, 1.4)	0.583
Solid foods with partial breastfeeding	0.5 (−0.1, 1.2)	0.125
Solid foods with formula feeding	1.1 (0.2, 2.0)	0.020

^a^ Adjusted difference in BMI at 6 years with respect to exclusively breastfed infants; * Analyses adjusted for sex, birth weight, and maternal education.

## 4. Discussion

In this current analysis, we found that infants who were provided solids foods in addition to formula feeding at 5 months of age had faster growth during infancy and up to 12 months of age compared to exclusively breastfed infants. Differences in weight were most pronounced between the ages of 2 to 6 months. Furthermore, the addition of solid foods among formula fed infants prior to the age of 6 months predicted greater BMI at 6 years.

The strength in this cohort lies in the longitudinal nature and the detailed information on infant feeding practices. In this way, we are able to describe dietary intake in infancy in greater detail than possible in many other studies in relation to BMI at 6 years of age. It is difficult to separate the effects of exclusive breastfeeding (and total duration of breastfeeding) from the introduction of solid foods at the age of 5 months. However, our findings are in line with existing evidence that show exclusively breastfed infants grow slower compared to infants of the same age who receive formula or solid foods [2,6] and that longer duration of breastfeeding may protect against later obesity potentially due to slower weight gain in infancy [6].

There are several factors that contribute to variations in infant feeding practices. Predictors of early introduction of solid foods include young maternal age, low maternal education and short (<4 weeks) duration of breastfeeding [17]. There were no significant differences with maternal age, and maternal education also did not appear to predict introduction of solid foods at 5 months of age, although the proportion of mothers with higher education was greater among the infants who were exclusively breastfed. We note that the mothers who started their infants on formula feeding had a higher mean BMI compared to mothers of exclusively breastfed infants, although the numbers are too few to detect a significant difference. There is evidence from epidemiological studies that women who are overweight or obese are less likely to breastfeed compared to normal weight women [18,19].

A probable explanation for the observed difference in growth rate between breastfed and formula fed infants is the relatively higher protein content of infant formula compared to breastmilk. High protein intake may have a stimulating effect on insulin-like growth factor 1 which can accelerate growth [20]. Furthermore, higher protein intake during infancy has been associated with faster weight gain and greater adiposity [21] and there is evidence that this is associated with higher BMI in childhood [4,7,22]. Another possible mechanism is that compared to breastmilk, infant formula contains a higher amount of omega-6 fatty acids which may play a role in promoting adipose tissue development and result in greater childhood adiposity [23].

Results from a randomized controlled trial performed in Iceland, showed no significant differences in energy intake [15], growth [14], or risk of being overweight [13] between those exclusively breastfed for 4 *vs*. 6 months. The reason may be a low amount of energy from solid foods [14,15] mainly consisting of infant cereals (67%) and median protein intake of only 0.9 g/day [14]. In other studies, exposing infants to solid foods prior to the age of 4 months was associated with being overweight or obese in early childhood [24,25], but it was suggested that the risk associated with the timing of introduction of solid foods might be greater among children who were no longer breastfed [24]. In the present study, we saw that partially breastfed infants who had been introduced to solid foods at the age of 5 months did not grow as fast as the infants who were formula fed and consuming solid foods. 

Together these findings suggest that in addition to the timing of complementary foods, the type of food, and the protein content of the infant formula introduced may influence infant growth [26]. In this cohort, 70% of infants who had been provided solid foods at 5 months were exclusively breastfed at 2 months of age. Mothers may introduce solid foods earlier if they find their infant appears hungry or worry that breast milk is inadequate for their infant’s needs [27]. However, limited data exists on the effects of specific food groups introduced during the complementary feeding period in relation to infant growth and childhood BMI. Further studies are needed in this area to determine whether rapid infant growth leads to children demanding more feedings or whether the introduction of formula feeding or solid foods is the main contributor.

Some limitations exist in this current analysis. The sample size is a possible limitation however, the thorough data on dietary intake and growth variables provides valuable information and the number is sufficient to analyze differences in infant feeding practices and its potential contribution to childhood weight status. Information on the exact timing of solid food introduction would have been useful as well as reasons affecting the duration of breastfeeding, particularly among the infants who had been provided solid foods at 5 months to better understand the observed association with BMI at 6 years. The aim of this present analysis was to determine the association between differences in infant feeding practices and childhood BMI, and even more detailed data related to diet and nutrient composition before the age of 5 months would have been beneficial. Overall, it appears that earlier formula feeding contributes to growth in infancy and adding solid foods along with formula feeding may influence growth in childhood compared to exclusively breastfed infants.

## 5. Conclusions

In this current analysis, we found that infants who were provided solid foods along with formula feeding prior to 6 months of age had faster growth during infancy and greater BMI at 6 years of age compared to exclusively breastfed infants. In a high-income country such as Iceland, early introduction of solid foods seems to further increase the risk of high childhood BMI of formula fed infants compared to exclusively breastfed infants. Furthermore, better breastfeeding promotion strategies may help reduce the incidence of childhood overweight. Further studies with more detailed information on infant feeding practices and statistical power are needed to determine the appropriate composition of infant formula and solid foods especially if mothers are for some reasons unable to breastfeed exclusively for 6 months.
